# Microwave-Assisted Production of Defibrillated Lignocelluloses from Blackcurrant Pomace via Citric Acid and Acid-Free Conditions

**DOI:** 10.3390/molecules29235665

**Published:** 2024-11-29

**Authors:** Natthamon Inthalaeng, Ryan E. Barker, Tom I. J. Dugmore, Avtar S. Matharu

**Affiliations:** Green Chemistry Centre of Excellence, Department of Chemistry, University of York, York YO10 5DD, UK; ni624@york.ac.uk (N.I.); ryan.barker@york.ac.uk (R.E.B.); tom.dugmore@york.ac.uk (T.I.J.D.)

**Keywords:** blackcurrant pomace, microwave hydrothermal treatment, defibrillated (ligno)cellulose, biomass pretreatment, citric acid pretreatment, biomass waste

## Abstract

Blackcurrant pomace (BCP) is an example of an annual, high-volume, under-utilized renewable resource with potential to generate chemicals, materials and bioenergy within the context of a zero-waste biorefinery. Herein, the microwave-assisted isolation, characterization and potential application of defibrillated lignocelluloses from depectinated blackcurrant pomace are reported. Depectination was achieved using citric acid (0.2–0.8 M, 80 °C, 2 h, conventional heating) and compared with acid-free hydrothermal microwave-assisted processing (1500 W, 100–160 °C, 30 min). The resultant depectinated residues were subjected to microwave-assisted hydrothermal defibrillation to afford two classes of materials: namely, (i) hydrothermal acid-free microwave-assisted (1500 W, 160 °C, 30 min; DFC-M1-M4), and (ii) hydrothermal citric acid microwave-assisted (1500 W, 160 °C, 30 min; DFC-C1–C4). Thermogravimetric analysis (TGA) revealed that the thermal stability with respect to native BCP (T_d_ = 330 °C) was higher for DFC-M1-M4 (T_d_ = 345–348 °C) and lower for DFC-C1–C4 (322–325 °C). Both classes of material showed good propensity to hold water but failed to form stable hydrogels (5–7.5 wt% in water) unless they underwent bleaching which removed residual lignin and hemicellulosic matter, as evidenced by ^13^C solid-state NMR spectroscopy. The hydrogels made from bleached DFC-C1–C4 (7.5 wt%) and bleached DFC-M1-M4 (5 wt%) exhibited rheological viscoelastic, shear thinning, and time-dependent behaviour, which highlights the potential opportunity afforded by microwave-assisted defibrillation of BCP for food applications.

## 1. Introduction

The utilization of unavoidable food supply chain wastes, a plentiful (ligno)cellulosic-rich renewable resource, has become an attractive proposition in the development of zero-waste biorefineries as we look to combat climate change via action through the United Nations 17 Sustainable Development Goals [1,2,3]. A biorefinery, which produces multiple products from a renewable feedstock, holds significant positive environmental promise, unlike petroleum refineries which are polluting, environmentally unfriendly and reliant on a finite, non-renewable resource [4,5,6].

Blackcurrant pomace (BCP), a large-volume byproduct of industrial harvesting and processing, primarily consists of cellulose, lignin, hemicellulose, pectin, polyphenols, fat and ash. The latter have been extensively studied for their bioactive properties, such as anti-oxidancy [7,8,9,10,11,12,13,14]. A few studies have investigated the potential to convert BCP into biofuels, e.g., bio-oil and biochar [15,16,17,18]. Alba et al. reported the use of pectin-free cellulosic matter as a source of dietary fibre [19,20]. However, regarding cellulosic material, BCP contains a complex of impurity mixtures as stated previously, posing challenges for its utilization in applications requiring cellulosic material. The content of impurities in BCP varies with lignin from 31 to 38%, hemicellulose from 14 to 30%, pectin from 10 to 16%, fats from 6 to 11%, ash from 3 to 4%, and polyphenols from 3.80 to 119.5 mg GAE/g sample [8,17,18,19].

Cellulose, the most abundant renewable polymer, is often regarded as a sustainable and long-lasting alternative to synthetic polymers. It offers numerous benefits such as cost-effectiveness, environmental friendliness, compatibility with living organisms, biodegradability, and its widespread use in various industries [21]. Cellulose can be isolated via chemical or mechanochemical pretreatment. Typically, cellulose is obtained through strong mineral acid hydrolysis to remove non-crystalline components and achieve a high crystalline structure [21,22,23,24,25,26]. However, the use of mineral acid creates a significant environmental hazard. Additionally, sugar dehydration products, such as furfural and 5-hydroxymethylfurfural (5-HMF), can form as by-products during the hydrolysis of sugars under harsh conditions. The presence of these compounds in hydrolysates acts primarily to inhibit microbial metabolism, negatively impacting biosynthesis processes. This inhibition can lead to substantial productivity losses and cost inefficiencies, and make industrial processes economically unfeasible [27].

Organic acid pretreatments have been widely used to replace mineral acids due to their efficiency, low cost, and less toxic by-products [28,29,30]. Citric acid (CA) is an example of a biobased renewable organic acid obtained from fermentation and has been utilised in various biomass pretreatment and extraction processes. It is effective in removing impurities such as lignin, hemicellulose, ash and inorganic matter from lignocellulosic feedstocks [29,31,32,33,34], facilitating the breakdown of fibre bundles into smaller units. The resulting cellulose, with its reduced impurities and improved fibre wetting, can be processed into a range of products such as paper, films, etc.

De Melo et al. demonstrated acid-free microwave hydrothermal fractionation of pectin and cellulose materials from biomass [35]. The industrial production of pectin uses hydrochloric acid (*p*Ka approx. −6) at 80 °C, which is highly corrosive and generates significant amounts of aqueous acidic waste. All traces of mineral acid need to be removed prior to pectin use in certain applications, for example, food and food packaging. The activation of lignocellulosic substances, such as BCP, can be achieved through step-by-step MHT, operated at temperatures ranging from 100 to 160 °C, followed by MHT reprocessing at 160 °C [36].

**Figure 1:**
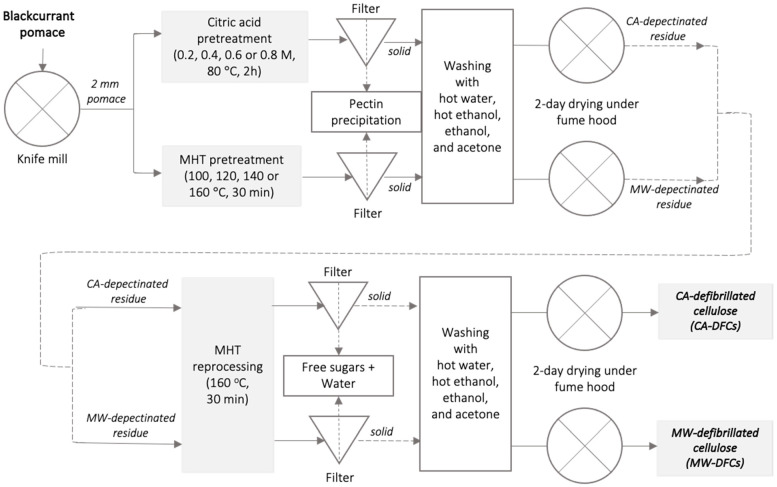
Schematic diagram of DFCs production from BCP via citric acid pretreatment and MHT pretreatment (Inthalaeng et al., 2023) [36].

Thus, herein, an alternative approach for production of defibrillated celluloses (DFCs) from BCP via CA pretreatment at various concentrations and reprocessing at 160 °C is reported. The citric acid-pretreated DFCs (CA-DFCs) were comparatively evaluated with the acid-free MHT-pretreated DFCs (MW-DFCs) obtained from our previous study [36] for the influence of pretreatment conditions, as summarized in Figure 1. The changes in the composition, structure, and morphology of CA-DFCs and MW-DFCs using various analytical techniques, including SEM, TGA, pXRD, and solid-state ^13^C CP/MAS NMR, are examined. The microwave processing temperatures used are based on existing knowledge of acid-free microwave-assisted pectin extraction and hemicellulose and cellulose decomposition temperatures [2,35,37,38]. Optimal pectin isolation is approx. 140 °C, and optimum cellulose decomposition is typically in excess of 190 °C, which leads to the formation of humins, chars and pseudo-lignins. Thus, restricting the upper temperature to 160 °C avoids decomposition of cellulose and ensures higher removal of hemicellulose. Therefore, defibrillated celluloses with higher cellulose content are envisaged. The specific aims of this study were (i) to examine and compare the effectiveness of citric acid and acid-free microwave-assisted extraction conditions at moderate temperatures on the extraction of lignocellulosic materials from BCP, with the goal to valorise food waste through a sustainable and green approach; (ii) assess the physicochemical properties of the resulting lignocellulose, including crystallinity, morphology, and chemical and structural compositions; (iii) evaluate the thermal and rheological properties to identify potential applications.

## 2. Results and Discussion

### 2.1. Effect of Pretreatment Conditions

**Figure 2:**
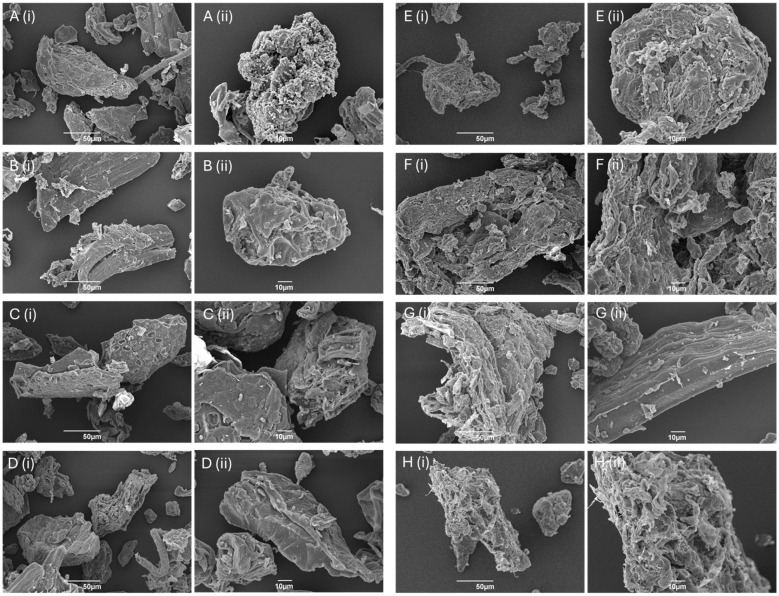
SEM images of defibrillated celluloses at magnification of (**i**) 500× and (**ii**) 1000×: (**A**) DFC-C1; (**B**) DFC-C2; (**C**) DFC-C3; (**D**) DFC-C4; (**E**) DFC-M1; (**F**) DFC-M2; (**G**) DFC-M3; (**H**) DFC-M4.

SEM was used to visualize the morphology of BCP (see Supplementary Materials Figure S6), CA-DFCs obtained after CA pretreatment at 0.2, 0.4, 0.6, or 0.8 M, followed by MW reprocessing at 160 °C (Figure 2A–D), and MW-DFCs derived after MHT at 100, 120, 140, or 160 °C, followed by MW reprocessing at 160 °C (Figure 2E–H). All isolated DFCs from BCP exhibited mainly bulk and rough pallet-like characteristics in different shapes and sizes. The CA-DFC surfaces appeared more open-like and smoother as the concentration of CA increased; however, the differences were not significant. This suggests that the degree of hydrolysis may not have varied much with change in CA concentration. Bondancia et al. found that cellulose isolated under shorter reaction times (1.5 h) with 65 wt% CA did not undergo complete hydrolysis [39]. The size and morphology of the cellulose derived from different CA conditions may be influenced by reaction time rather than the concentration of CA. Interestingly, although Fouad et al. and Trache et al. suggest a link between smooth surfaces with loss of hemicellulosic and lignin components, detailed IR studies of the fibres pre- and post-treatment (see Supplementary Materials Figure S1) revealed no significant correlation [40,41].

CA-DFCs revealed a smoother surface and smaller sizes (11–70 μm) compared to MW-DFCs (18–130 μm). The smaller particle size observed in CA-DFCs may be due to the breakdown of the internal structure of cellulose, in particular the amorphous regions, during acid hydrolysis in the CA pretreatment step. On the other hand, MW-DFCs exhibited a flaky surface and uneven bundle structure. This rough and flaky surface is primarily a result of the defibrillation process induced by chemical treatment [25,42].

**Figure 3:**
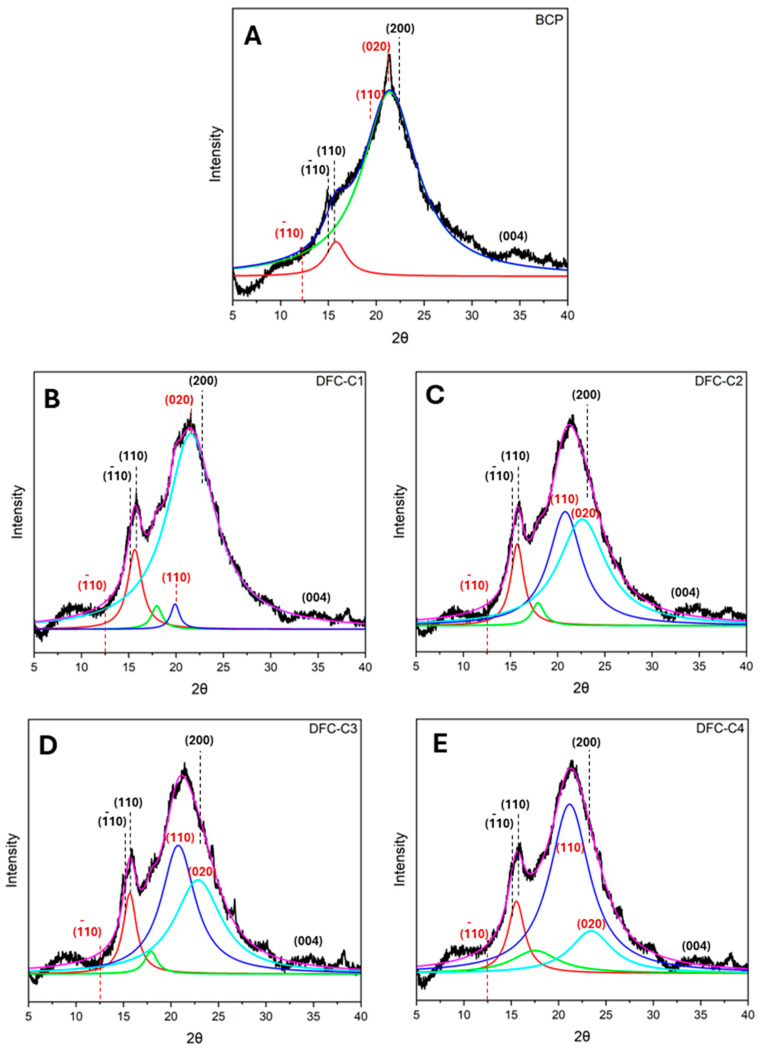

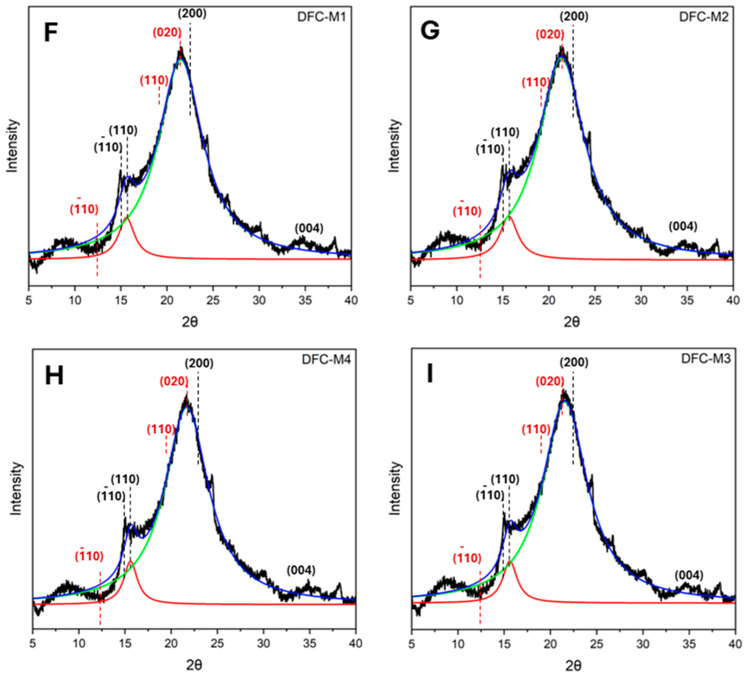
X-ray diffractograms of (**A**) blackcurrant pomace (BCP); (**B**–**E**) defibrillated celluloses obtained from citric acid pretreatment (CA-DFCs; C1–C4 corresponded to citric acid concentrations of 0.2–0.8 M, respectively); (**F**–**I**) defibrillated celluloses obtained from MW pretreatment (MW-DFCs; M1–M4 corresponded to MW pretreatment temperature of 100–160 °C, respectively). Black dash lines and red dash lines refer to crystalline plane of cellulose I and II, respectively, based on French et al., 2014 [46].

The XRD diffractogram of native BCP (Figure 3A) showed a primary broad peak with high intensity between 20° and 22°, a secondary broad peak between 15° and 17°, and a low-intensity peak at 34.5°, reflecting a predominantly cellulosic composition [43,44]. This is also observed in various biomass types, such as coffee husk, corn cob, teff straw and sweet sorghum stalk [45]. After subjecting BCP to CA pretreatment followed by MHT reprocessing, the resulting CA-DFCs (Figure 3B–E) showed a change in their XRD patterns. The characteristic peaks observed at approximately 15°, 16°, 20.1°, and 21.5° are indicative of ($\bar{1}$10, (110) of cellulose I, and (110), (020) of cellulose II, respectively. However, the peak at 12.5°, corresponding to ($\bar{1}$10) of cellulose II, was not observed [44,46,47,48]. On the other hand, MW-DFCs (Figure 3F–I) exhibited XRD patterns similar to that of native BCP cellulosic material. These findings suggest citric acid pretreatment modifies the structure of cellulose more than microwave pretreatment. However, this observation is inconsistent with the literature, which reported that citric acid treatment did not alter the crystalline structure of cellulose [49,50].

Lorentzian deconvolution was employed to examine the crystalline pattern of BCP-defibrillated cellulose. Upon pretreatment of BCP with citric acid, the intensity of the diffraction peak of the (110) plane of cellulose I in CA-DFCs (Figure 3B–E) was found to be higher compared to the XRD pattern of untreated BCP. With increasing citric acid concentration, the crystalline plane (110) of cellulose II was observed to be shifted to the (020) plane. This indicated that citric acid contributes to enhancing the change in the crystalline plane of cellulose. However, the crystalline plane of MW-DFCs (Figure 3F–I) remained unchanged compared to native BCP, indicating that the crystalline structure of cellulose is not affected by microwave treatment.

Regarding the Segal crystallinity index (*CI*), native BCP exhibited a *CI* of 38.8%, while all DFC samples showed an increase in *CI* after pretreatment, ranging from 42.4 to 56.0% (see Supplementary Materials Table S1). This suggests that CA pretreatment together with MHT reprocessing and double MHT processing induced the changes in the crystallinity index of BCP. However, the *CI* of CA-DFCs remained consistent between 43.0% and 44.2%, suggesting that varying CA concentrations prior to MHT reprocessing have a minimal impact on the cellulose crystallinity index. In contrast, varying MW temperatures before MHT reprocessing significantly enhanced crystallinity, with the *CI* reaching up to 56.0%.

**Figure 4:**
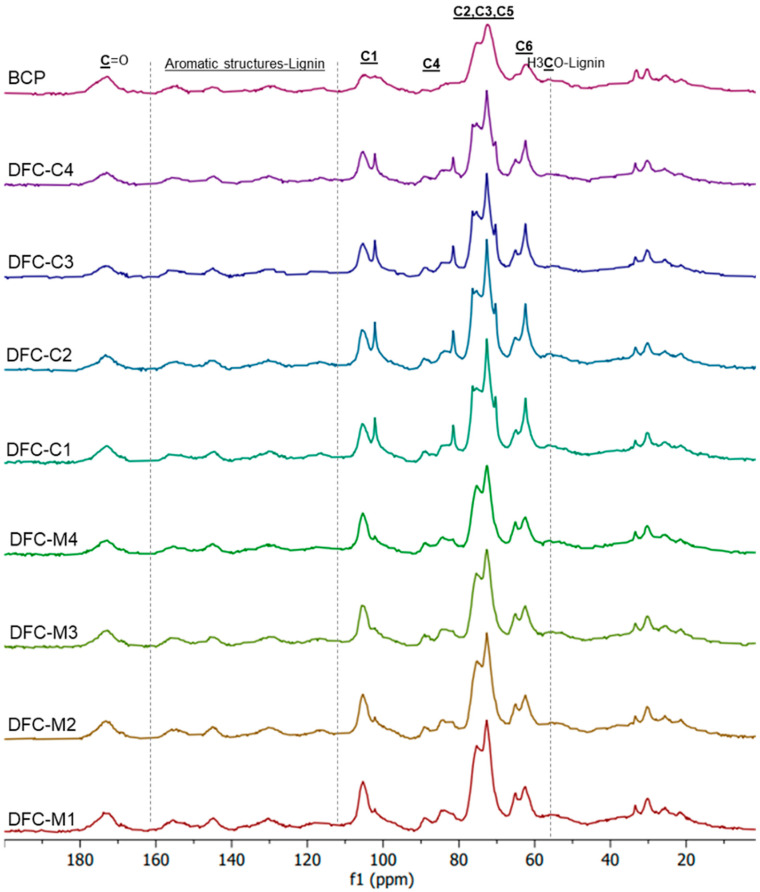
Solid state ^13^C CP/MAS NMR spectra of blackcurrant pomace (BCP), defibrillated celluloses obtained from citric acid pretreatment (CA-DFCs; C1–C4 corresponded to citric acid concentrations of 0.2–0.8 M, respectively), and defibrillated celluloses obtained from MW pretreatment (MW-DFCs; M1–M4 corresponded to MW pretreatment temperature of 100–160 °C, respectively).

The cellulose I and II structures of DFCs were further confirmed by ^13^C CP/MAS NMR spectroscopy (Figure 4). All spectra revealed the presence of C1–C6 of cellulose signals, observed within the range of 60 to 120 ppm. Additionally, signals observed in the range of 20 to 35 ppm suggested potential evidence of the aliphatic region [35,51,52,53]. The signals at 56 ppm and 174 ppm may be attributed to the methoxy (-OCH_3_) group and carbonyl (C=O) group, respectively, in ester bonds of lignin regions [51]. The aromatic signals of lignin ranging from 110 to 160 ppm were detected [54].

The untreated BCP and MW-DFCs presented an NMR pattern of cellulose I. However, a different pattern emerged in CA-DFCs. There was a splitting peak observed for C1 (at 102 and 105 ppm) and C4 (at 82, 84, and 89 ppm), along with four sub-peaks in the regions of C2, C3, and C5. This difference might be explained by a partial conversion of cellulose I to cellulose II. The characteristic four sub-peaks in the C2, C3, and C5 regions appeared along with the presence of a peak at C6 (62 ppm) resembling cellulose II morphology [55,56]. The characteristic peak of C6 for cellulose I (at 65.8 ppm) was also present [57], suggesting that the cellulose underwent a partial conversion rather than a complete transformation from cellulose I to cellulose II during the CA pretreatment step.

Another possibility is the folding of xylan structures occurring during the treatment, as evidenced by the presence of peaks at 105, 72, 75, 82, and 64 ppm, corresponding to Xn1 to Xn5 of twofold xylan, respectively. In addition, the Xn1 to Xn5 signals of threefold xylan appeared at 102, 73, 74, 77, and 63 ppm, respectively [58]. Twofold xylans can be bound with cellulose microfibrils, which effectively extend the crystalline region in the cellulose system, resulting in better resistance to the microbial hydrolysis process [58]. On the other hand, self-assembled crystal hydrate xylans can readily form in aqueous solution, in which threefold xylans may occur in crystalline hydrate form [59]. It can be seen that the folded xylans signals are prominent in CA-DFCs. This may be explained by the treatment of BCP in the first step with citric acid, which can remove sidechain uronic acids of xylans, resulting in less solubility of xylan in water, making it readily self-assembled into twofold, threefold xylans or xylan hydrate form. The effect of the xylan sidechains on the formation of xylan hydrate crystals has been explained by Johnson et al. [59]. The presence of xylose in carbohydrate analysis can further confirm the presence of xylans in DFC samples.

Lignin and carbohydrate analyses of native BCP and defibrillated cellulose samples (DFCs) were conducted to evaluate the impact of pretreatment on removing amorphous structures (see Supplementary Materials Figures S2 and S3). Both CA-DFCs and MW-DFCs displayed elevated levels of glucose, compared to untreated BCP. This increase in glucose suggests that impurities such as hemicellulose, pectin, and lignin were partially removed during pretreatment, leading to a higher observed cellulose content. Moreover, MW-DFCs exhibited lower concentrations of xylose and galacturonic acid, monomers of hemicellulose and pectin, respectively, compared to CA-DFCs. This observation indicates that double MHT processing may be more effective in breaking down hemicellulose and pectin in lignocellulosic biomass than the citric acid-MHT process, likely due to enhanced thermal degradation. However, lignin content seems slightly increased in both CA-DFCs and MW-DFCs. This can be explained by the complexity of lignin structure, which makes it resistant to degradation during these processes, whereas other components, such as pectin and hemicellulose, are more susceptible to degradation. Additionally, it is possible that pseudo-lignin from 5-hydroxymethyl furfural (5-HMF) and furfural formed at high microwave processing temperatures, which can be observed in microwave-assisted production of defibrillated cellulose from other biomass sources [2,35,37].

### 2.2. Physical Properties of Defibrillated Celluloses

#### 2.2.1. Thermal Behaviour

**Figure 5:**
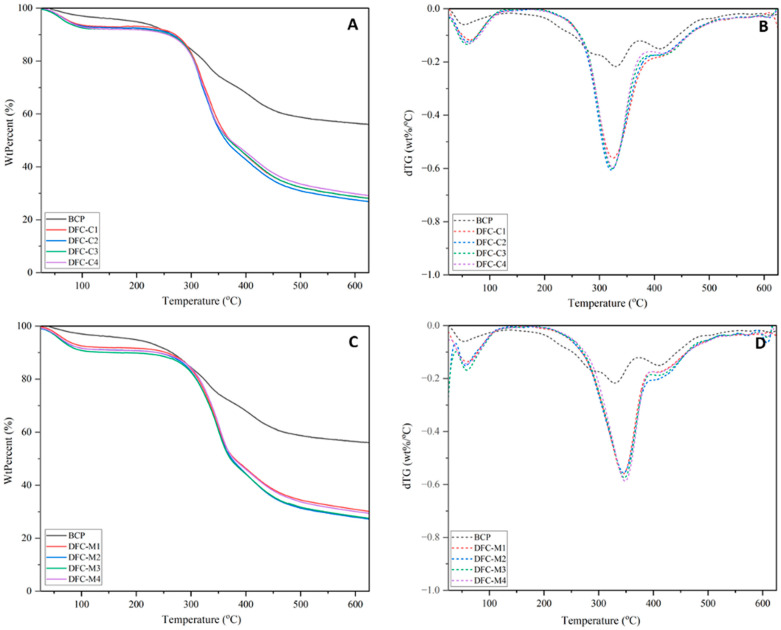
TGA thermogram of (**A**) defibrillated celluloses obtained from citric acid pretreatment (CA-DFCs; C1–C4 corresponded to citric acid concentrations of 0.2–0.8 M, respectively) and (**C**) defibrillated celluloses obtained from MW pretreatment (MW-DFCs; M1–M4 corresponded to MW pretreatment temperature of 100–160 °C, respectively). dTG of (**B**) CA-DFCs; C1–C4 and (**D**) MW-DFCs; M1–M4.

Comparing the thermal behaviour of MW-DFCs, obtained through acid-free MHT, with CA-DFCs produced through CA pretreatment revealed that the former (MW-DFCs) exhibited higher thermal stability (see Figure 5). The derivative thermograms (dTGs) shown in Figure 5B,D further illustrate the maximum degradation temperature (T_d_). For CA-DFCs, the T_d_ decreased with increasing CA concentration. Specifically, the T_d_ values for CA-DFCs (DFC-C1: 325 °C, DFC-C2: 322 °C, DFC-C3: 324 °C, and DFC-C4: 325 °C) were 5 °C lower compared to untreated BCP (330 °C).

In contrast, MW-DFCs exhibited T_d_ values (DFC-M1: 345 °C, DFC-M2: 346 °C, DFC-M3: 347 °C, and DFC-M4: 348 °C), approximately 15 °C higher than that of untreated BCP. This suggests that the MW pretreatment for pectin removal has less impact on cellulose structure, preserving its thermal stability [35]. These findings align well with studies on defibrillated cellulose obtained from various sources via acid-free MHT, where T_d_ values ranged from 340 to 374 °C [2,35,60]. Additionally, the presence of lignin, as evidenced in Figure 4, contributes to increased thermal stability [61].

#### 2.2.2. Water-Holding Capacity (WHC) and Hydrogel Formation

The WHC and hydrogel formation capability of the defibrillated celluloses are summarized in Table 1. The WHC values of CA-DFCs and MW-DFCs were distinct (*p* < 0.05), however, not significantly different in the same group. The highest WHC was observed in CA-DFCs (5.38–5.60 g/g), followed by MW-DFCs (4.77–5.00 g/g). These values are below those obtained from defibrillated celluloses derived from orange peel and pea waste using acid-free MHT [35,60]. This implies that the DFCs produced from BCP through both CA and MHT pretreatments may not have the same water-holding capacity. This difference in water-holding ability may be attributed to the elevated lignin content present in the resulting DFCs, as evidenced in solid state ^13^C CP/MAS NMR spectra and TGA thermograms (Figure 4 and Figure 5). The ability to form hydrogels was tested using the same method detailed by Inthalaeng et al. at various concentrations of DFCs in deionized water (2.5, 5, 7.5, and 10 wt%) [36]. All CA-DFCs and MW-DFCs derived from BCP did not afford a stable hydrogel at any concentration, which may be due to the residual lignin present. Thus, alkali bleaching of both CA-DFCs and MW-DFCs resulted in the formation of stable hydrogels at a concentration of 5 wt%, with the CA-DFCs gelling at 7.5 wt%. Interestingly, the NMR of the DFC-C series revealed more folded xylans, whilst the DFC-M series revealed less rigid xylan structures. The latter show propensity to form gels at 5 wt% in water, whilst the former form gels at 7.5 wt% in water. Thus, it appears that cellulose with less rigid xylans is more favourable for hydrogel formation.

#### 2.2.3. Rheological Studies of Hydrogels

**Figure 6:**
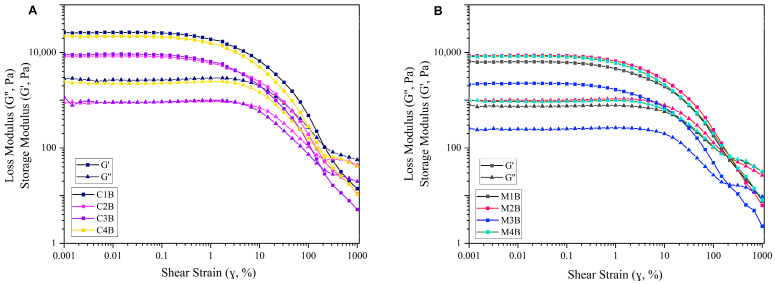
Amplitude sweeps of defibrillated cellulose hydrogels; (**A**) at 7.5 wt% of bleached CA-DFCs; C1B, C2B, C3B and C4B; (**B**) at 5 wt% of bleached MW-DFCs; M1B, M2B, M3B and M4B.

Figure 6 displays the rheological amplitude sweep curves of the hydrogels derived from 7.5 wt% of bleached DFC-C1–C4 and 5 wt% of bleached DFC-M1–M4. Within the initial phase of small shear strain, the hydrogels exhibited a linear viscoelastic region (LVR). During this region, all hydrogels displayed solid-like viscoelastic behaviour (G′ > G″), indicating that the hydrogel behaved more like a solid than a fluid, with elastic behaviour dominating over viscous behaviour. As the shear strain increased, both G′ and G″ decreased, suggesting that the hydrogel network structure underwent deformation and then reached a crossover point or flow point (G′ = G″) at a shear strain between 147% and 316%. Past the crossover point, the G″ value became higher over G′, indicating that the hydrogel began to exhibit fluid-like behaviour [62]. The yield point, marking the point at which G′ begins to decrease, was found at a shear strain of 0.1% and was comparable between both classes of materials. The ability of cellulose to form gels depends on the degree of fibrillation and surface charge; the increase in both fibrillation and specific surface area can enhance the water-holding capacity of fibrillated cellulose [63,64]. However, G′ for DFC-C1–C4 was higher than that of DFC-M1–M4, maybe due to the higher concentration of the former, resulting in increased gel strength in the hydrogel. The hydrogels made from bleached DFC-C1–C4 (7.5 wt%) and bleached DFC-M1-M4 (5 wt%) exhibited rheological behaviour similar to that of high-consistency enzymatic fibrillated cellulose (HefCel) gel in which the LVR and yield point occurred at shear strains below 1%, and the flow point was observed beyond 100% strain [65]. Additionally, HefCel cannot form a stable gel at concentrations below 7% [65].

**Figure 7:**
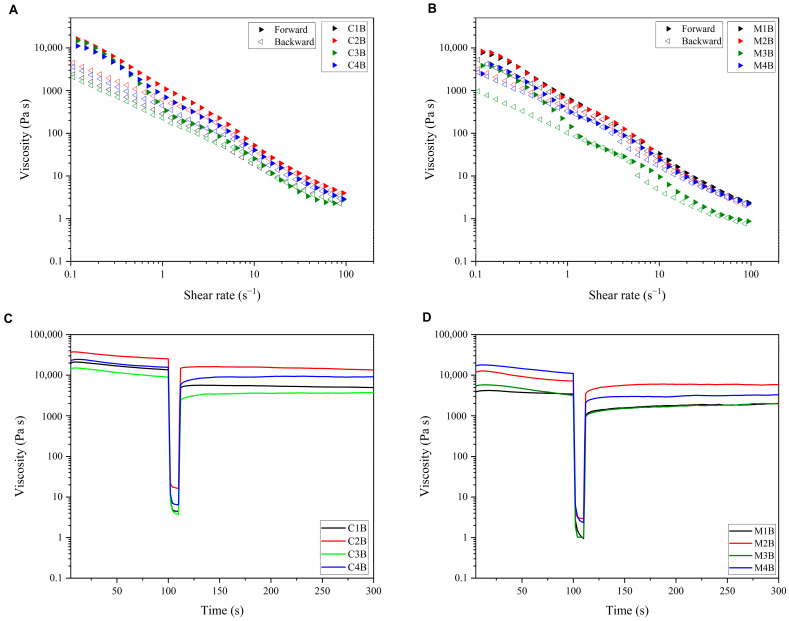
Flow curves with increasing and decreasing shear rate of defibrillated cellulose hydrogels: (**A**) at 7.5 wt% of bleached CA-DFCs; C1B, C2B, C3B and C4B; (**B**) at 5 wt% of bleached MW-DFCs; M1B, M2B, M3B and M4B, and thixotropy test of (**C**) 7.5 wt% of bleached CA-DFCs; C1B, C2B, C3B and C4B; (**D**) 5 wt% of bleached MW-DFCs; M1B, M2B, M3B and M4B.

Flow and thixotropic analyses were performed to investigate the flow behaviour and time-dependent structural changes in bleached DFC hydrogels, presented in Figure 7. The viscosity of all bleached DFC samples decreases with increasing shear rate, indicating shear thinning behaviour. This phenomenon is linked to the network of microfibrillated cellulose disruption under shear action, leading to the aggregation or breakdown of the system and subsequent reduction in viscosity [66]. In addition, subsequently reducing the shear rate allows the network to reform, resulting in an increase in viscosity, and a hysteresis loop within the overall flow curve was observed (Figure 7A,B), indicating thixotropic behaviour in bleached DFC hydrogel samples derived from blackcurrant pomace. These behaviours have also been found in microfibrillated suspensions of pea hull fibre [67]. However, we note a slightly unusual hysteresis loop showing crossover for sample M3B (Figure 7B), which may be due to the presence of an artefact in the sample, for example, microscopic particulate matter.

The thixotropy test was conducted to monitor viscosity and structural changes of bleached DFC hydrogels over time. In Figure 7C,D, it can be seen that all bleached DFC hydrogels gradually decreased with time at a shear rate of 0.1 s^−1^, and a deep drop was observed with the application of a high shear rate (100 s^−1^). Then, the bleached DFC hydrogels showed increased viscosity with time, representing the time-dependent behaviour. However, hydrogels derived from bleached MW-DFCs showed lower time-dependent behaviour than bleached CA-DFCs. This again can be explained by the difference in concentration of bleached DFCs in hydrogels, in which 5 wt% MW-DFCs and 7.5 wt% CA-DFCs were prepared, resulting in a stronger and more viscous network structure in bleached CA-DFCs. Both types of bleached DFCs can be considered for industrial food applications, as they show reformed structures over time and can be applied as both functional and nutritional substances.

## 3. Materials and Methods

### 3.1. Materials

Blackcurrant pomace (BCP) was provided by Lucozade Ribena Suntory Ltd., Hayes, UK and was used as feedstock for DFCs production. The as-received BCP was dried (ambient condition, 7 days), milled (≤2 mm) and stored in air-tight bags. The MW-DFCs were prepared as previously reported [36], and are labelled as DFC-M1, DFC-M2, DFC-M3, and DFC-M4, corresponding to MW pretreatment at temperatures of 100, 120, 140, and 160 °C, respectively.

### 3.2. Blackcurrant Pomace and DFCs Characterisation

Proximate and ultimate analyses of BCP were determined based on the standard NREL method [68] as follows: BCP after pretreatment or DFC samples (100 mg, denoted as W) were mixed with 72% H_2_SO_4_ (1 mL) and subjected to shaking in a water bath (40 °C, 2 h). Thereafter, the mixtures were supplemented with deionized water (28 mL) to make a final concentration of 4% H_2_SO_4_ and then autoclaved (121 °C, 1 h). The resulting hydrolysed sample was filtered through a vacuum filter crucible (denoted as W_0_). The resulting liquid was then subjected to carbohydrate analysis using HPLC-RID, which operated on an Agilent 1260 equipped with an Agilent Hi-Plex H+ column (300 × 7.7 mm, 8 μm particle size) (Agilent Technologies, Santa Clara, CA, USA). The analytical conditions included a reverse-phase system with 0.005 M H_2_SO_4_ as the mobile phase, an injection volume of 5 μL, a flow rate of 0.4 mL/min, and column and detector temperatures set at 60 °C and 55 °C, respectively. The total runtime for this procedure was 30 min.

Following this, the solid residues were rinsed with distilled water and subsequently dried in an oven at 105 °C overnight. After cooling within a desiccator, the weight of the crucible containing the solid residue was recorded as W_1_. The calculation of lignin content was performed using the following equation:

$$Klason\;lignin\left(\%\right)=\frac{W_{1}-W_{0}}{W}\times 100$$

Chemical and elemental composition of BCP is given in Table 2.

### 3.3. Production of CA-DFCs

BCP (40 g) was pretreated to remove pectin with aqueous CA solution (200 mL) at varying concentrations (0.2, 0.4, 0.6 or 0.8 M, 80 °C, 2 h). The mixture was filtered, and the resulting depectinated BCP residues were subsequently washed successively with hot water (90–95 °C), hot ethanol (60–65 °C), ethanol, and acetone, and were then air-dried under a fume hood (2 days). The dried depectinated BCP (20 g) was combined with deionized water (300 mL) and subjected to microwave irradiation (Milestone Synthwave Microwave, 1500 W, 160 °C, 30 min; with a 15 min ramp and a 15 min hold). Thereafter, the resultant mixture was filtered (Buchner, Bayview Ave, ON, Canada), and the crude CA-DFCs were washed with hot water, hot ethanol, ethanol, and acetone. The washed CA-DFC residues were air-dried under a fume hood (2 days), manually ground, sieved (250 µm), and designated as DFC-C1, DFC-C2, DFC-C3, and DFC-C4, corresponding to citric acid pretreatment at concentrations of 0.2, 0.4, 0.6, and 0.8 M, respectively.

### 3.4. Scanning Electron Microscopy (SEM)

A small quantity of the DFC samples was applied onto a carbon tab affixed to an SEM stub. Subsequently, the SEM stubs underwent sputter coating with a 5 nm layer of gold/palladium using a Polaron SC7640 sputter coating apparatus (Quorum Technologies Ltd., East Sussex, UK). The specimens were then visualized using a Jeol JSM 6490LV scanning electron microscope, which operated at 5 Kv (JEOL Ltd., Tokyo, Japan).

### 3.5. Solid State ^13^C CP/MAS NMR Spectroscopy

^13^C CP/MAS NMR analysis was performed following the modified literature procedures [35]. Sample spectra were acquired using a Bruker Avance III HD spectrometer (400 MHz) equipped with a 4 mm H(F)/X/Y triple-resonance probe and 9.4T ascend superconducting magnet (Bruker, Bremen, Germany). The spinning rate was set at 20 kHz, with a contact time of 1 ms and a recycle delay of 8 s. A total of 8500 scans were accumulated. The spectra were analysed using MestReNova ×64 software version 14.3.1.31739.

### 3.6. Powder X-Ray Diffraction (pXRD)

pXRD analysis of the DFC samples was conducted using the Panalytical Aeris powder X-ray diffractometer. This instrument employed a Beta nickel source filter and operated with a scan speed of 0.2°/s at room temperature. The samples were scanned within a range of 2θ = 5–40° [60]. The spectra were plotted using Origin software version 2022bSr1, and peaks were deconvoluted through Lorentzian fitting function. The crystallinity index (*CI*) of native BCP, BCP after pretreatment, and DFC samples was calculated using the Segal equation [69]:

$$CI\;\left(\%\right)=\frac{I_{t}-I_{a}}{I_{t}}\times 100$$

where *I_t_* represents the total intensity of the major crystalline peak at 2θ = 22.7° for cellulose *I*, and 21.7° for cellulose II, and *I_a_* is the intensity of amorphous region at 2θ = 18° for cellulose *I*, and 16° for cellulose II. The CIs were calculated from the baseline subtracted curve fit using Origin software version 2022bSr1.

### 3.7. Thermogravimetric Analysis (TGA)

TGA of the DFC samples was carried out using the Stanton Redcroft STA625 (Stanton Instruments Ltd., London, UK). In this process, DFC samples (10 mg) were placed into an aluminium pan and compared against an empty reference aluminium pan while being exposed to a nitrogen gas atmosphere. The temperature was incrementally raised from 25 °C to 625 °C at a rate of 10 °C/min [60]. The resulting data were then analysed using Origin software version 2022bSr1.

### 3.8. Water-Holding Capacity (WHC)

The determination of WHC was conducted following the modified literature procedures [70,71]. The samples (0.2 g) were suspended in deionized water (10 mL) within a centrifuge tube. Subsequently, the mixture was agitated using a vortex mixer (2000 rpm, 1 min), subjected to sonication (30 °C, 20 min), and allowed to stand overnight (room temperature). Afterward, centrifugation was performed (3900 rpm, 20 min), and the liquid fraction was carefully decanted. WHC was determined by using the following equation:

WHC = [(Weight of wet sediment − Weight of tube)/Weight of sample] × 100

### 3.9. Hydrogel Formation

DFC samples were placed in deionized water (2.5 mL) at concentrations of 5% and 7.5% w. Subsequently, the mixture was vortexed (2000 rpm, 1 min) and sonicated (30 °C, 20 min). The mixtures were left undisturbed (room temperature, 18 h), and the stability of the hydrogel was assessed by qualitatively evaluating gel strength through the inversion of the gel vial for 30 min.

### 3.10. Rheology Studies

Amplitude sweep tests of hydrogels were conducted using a stress-controlled rheometer (Anton Paar Physica, MCR-301 rheometer, Graz, Austria) equipped with a serrated parallel-plate measuring system (25 mm diameter, 1 mm gap) at room temperature (25 °C), with an angular frequency (ω) of 10 rad/s and amplitude strain (γ) ranging from 0.001% to 1000%. Data points were collected at a frequency of 6 points per decade, resulting in a total of 37 measuring points [72]. To conduct this test, stable hydrogels were prepared from bleached DFC-C1–C4 (7.5 wt%) and bleached DFC-M1-M4 (5 wt%). These hydrogels were subjected to increasing shear strain (γ, %) while maintaining a constant angular frequency. The relationship between the increasing shear strain and the storage modulus (G′) and loss modulus (G″), which describes the solid-like and liquid-like viscoelastic behaviour of the hydrogel, respectively, was plotted, and the linear viscoelastic region (LVR) of each sample was identified.

Flow and thixotropic analyses of bleached DFCs hydrogels were conducted with a Kinexus rheometer (Malvern Instruments Ltd., Malvern, UK) equipped with a smooth-plate measuring system (20 mm diameter, 1 mm gap) at 25 °C. Flow curves were measured from 0.1 to 100 s^−1^ (forward) and then 100 to 0.1 s^−1^ (backward) in 2 min. Thixotropic tests were conducted by step test while applying a shear rate of 0.1 s^−1^ for 100 s, followed by shearing at a rate of 100 s^−1^ for 10 s and, finally, at a 0.1 s^−1^ rate for 5 min to observe the viscosity recovery.

### 3.11. Attenuated Total Reflection Infrared (ATR-IR) Spectroscopy Analysis

The IR spectra of BCP after pretreatment and DFC samples were recorded between 650 and 4000 cm^−1^ with 4 scans and force gauge between 100 and 120 using a Perkin Elmer Spectrum 400 IR (Perkin Elmer, Waltham, MA, USA).

### 3.12. Statistical Analysis

Statistical analysis, including analysis of variance (ANOVA) and Fisher’s least significant difference (LSD) test, was performed using IBM SPSS Statistics software version 28.0.1.1. The significance level was set at *p* < 0.05 with a confidence interval of 95%. WHC was replicated three times, while solid state ^13^C CP/MAS NMR, TGA, and pXRD were conducted with a single replication.

Detailed analysis of changes in lignocellulosic composition and sugar analysis of pre- and post-treatment lignocellulosic materials are given in Supplementary Materials.

## 4. Conclusions

The valorisation of BCP, particularly for bioactive compounds, has been well explored in the literature. However, research focused on valorisation of the cellulosic materials in BCP remained limited. In this study, we demonstrate that BCP is a potential renewable feedstock for the production of defibrillated celluloses, derived from acid-free microwave-assisted and/or citric acid processing. These two approaches yield lignocellulosic materials with different physicochemical properties, in which CA-DFCs exhibited smaller size, lower crystallinity and lower thermal stability compared to MW-DFCs. The pulp yields (see Supplementary Materials Table S1 were higher for microwave treatment (75–85 wt%) compared with citric acid treatment (60–61 wt%). The latter is more destructive to the (ligno)cellulosic structure of BCP and reorganises the cellulosic structure (increasing the concentration of citric acid gives a cellulosic structure with greater crystalline cellulose 110 content). Microwave treatment alone had little to no effect on the redistribution of crystalline cellulose 200 content compared to the native BCP.

Interestingly, the folded xylans, identified via CA-DFCs by ^13^C CP/MAS NMR and residue xylose via carbohydrate analysis, suggest that citric acid hydrolysis of sidechain xylans leads to their reduced solubility in water, producing self-assembled structures. This finding supports the idea that microwave-assisted pretreatment at moderate temperatures (100–160 °C) can effectively prevent the self-assembly of xylans, which is advantageous for the fractionation of carbohydrate polymers in biomass.

Both processing methods produced DFC materials that have the ability to hold water, with the bleached DFC samples exhibiting hydrogel-forming abilities. The viscoelastic, shear-thinning and time-dependent behaviours observed in bleached DFC gels indicate their potential for various food applications. This research contributes to the development of zero-waste biorefineries, and avoidance of harsh mineral acids lessens the environmental footprint. However, the prospective applicability hinges on a thorough techno-economic analysis that explores hydrogel stability, rheology, and characterisation with counterpart materials (e.g., straw) and technologies (e.g., liquid hot water) in either the literature or commercial arena. As part of future work, it would be interesting to investigate the effect of enzymatic pretreatment on the production of defibrillated celluloses and its direct comparison, HefCel.

## Figures and Tables

**Table 1:** Water-holding capacity and hydrogel formation capability of DFCs.

Scheme	WHC (g/g) *	Hydrogel Formation
DFC-C1	5.42 ± 0.40 ^a^	Formed at 7.5 wt% for bleached sample
DFC-C2	5.60 ± 0.31 ^a^	
DFC-C3	5.38 ± 0.45 ^a^	
DFC-C4	5.48 ± 0.14 ^a^	
DFC-M1	4.77 ± 0.14 ^b^	Formed at 5 wt% for bleached sample
DFC-M2	5.00 ± 0.24 ^b^	
DFC-M3	4.89 ± 0.24 ^b^	
DFC-M4	4.91 ± 0.10 ^b^	

* Values are displayed as mean ± standard deviation (*n* = 3) and distinct superscript letters (^a,b^) indicate statistically significant differences (*p* < 0.05).

**Table 2:** Chemical and elemental composition of BCP.

Content	Value (wt%)
Moisture ^a^	7.57 ± 0.17
Ash ^a^	2.46 ± 0.17
Protein ^b^ (N × 6.25)	10.66 ± 0.31
Cellulose ^a^	8.74 ± 0.52
Lignin ^a^	46.80 ± 2.61
C ^b^	48.43 ± 0.13
H ^b^	5.78 ± 0.04
N ^b^	1.71 ± 0.05
Remainder ^b,c^	44.30 ± 0.15

^a^ Triple replication. ^b^ Double replication. ^c^ May contain O and S.

## Data Availability

The original contributions presented in the study are included in the article/Supplementary Materials, further inquiries can be directed to the corresponding author.

## Supplementary Materials

The following supporting information can be downloaded at: https://www.mdpi.com/article/10.3390/molecules29235665/s1, Figure S1: ATR-IR spectra of BCP residues after citric acid pretreatment and MW pretreatment, and defibrillated cellulose (CA-DFCs and MW-DFCs).; Figure S2: Carbohydrate and sugar analysis of BCP residues after citric acid pretreatment and MW pretreatment, and defibrillated cellulose (CA-DFCs and MW-DFCs).; Figure S3: Klason lignin analysis of native BCP, BCP residues after citric acid pretreatment and MW pretreatment, and defibrillated cellulose (CA-DFCs and MW-DFCs).; Figure S4: X-ray diffractograms of BCP residues after citric acid pretreatment and MW pretreatment.; Figure S5: Thermogravimetric analysis of BCP, BCP residues after citric acid pretreatment and MW pretreatment.; Figure S6: SEM images of BCP.; Table S1: Crystallinity index of native BCP, BCP after pretreatment and DFC samples, and pulp yields.

## Abbreviations

BCP: Blackcurrant pomace; CA, Citric acid; CA-DFCs, citric acid-pretreated defibrillated (ligno)celluloses; DFC, defibrillated (ligno)cellulose; MHT, microwave hydrothermal treatment; MW, microwave; MW-DFCs, microwave-pretreated defibrillated celluloses.
