# Supplementary material for: Microwave-Assisted Production of Defibrillated Lignocelluloses from Blackcurrant Pomace via Citric Acid and Acid-Free Conditions
Source: Molecules. 2024 Nov 29;29(23):5665. doi: 10.3390/molecules29235665 (PMC11643517; doi:10.3390/molecules29235665)
Supplement: Supplementary file 1 [file molecules-29-05665-s001.zip › molecules-3217744-supplementary.pdf]

Supporting information

Microwave-Assisted Production of Defibrillated Lignocelluloses from Blackcurrant Pomace via Citric Acid and Acid-Free Conditions

Natthamon Inthalaeng, Ryan E. Barker, Tom I. J. Dugmore and Avtar S. Matharu *

Green Chemistry Centre of Excellence, Department of Chemistry, University of York, York YO10 5DD, UK; ni624@york.ac.uk (N.I.); ryan.barker@york.ac.uk (R.E.B.); tom.dugmore@york.ac.uk (T.I.J.D.)

* Correspondence: avtar.matharu@york.ac.uk

Table S1. Crystallinity index of native BCP, BCP after pretreatment and DFC samples, and pulp yields.

	CI of Native BCP (%)	*CI of BCP after pretreatment (%)*	*CI of DFCs (%)*	*Pulp Yield (wt%)*
Native BCP	38.8			
DFC-C1		32.4	43.1	61
DFC-C2		41.5	43.5	61
DFC-C3		34.9	44.2	61
DFC-C4		39.0	43.0	60
DFC-M1		39.9	47.2	75
DFC-M2		38.%	42.4	80
DFC-M3		36.6	51.1	80
DFC-M4		43.6	56.0	85

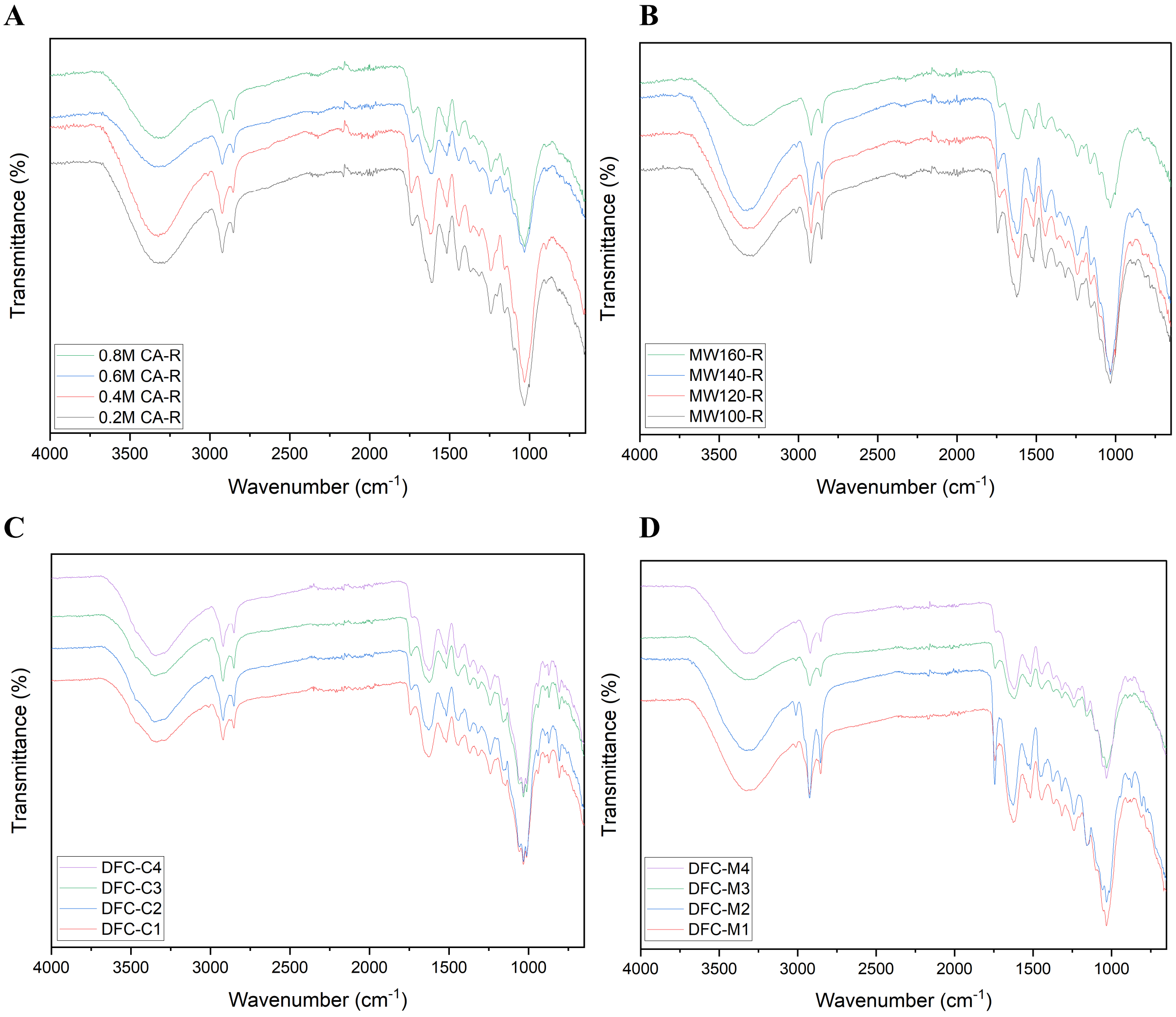

Fig. S1. ATR-IR spectra of (**A**) BCP residues after citric acid pretreatment with concentrations of 0.2–0.8M, respectively; (**B**) BCP residues after MW pretreatment with MW temperature of 100–160 °C, respectively; (**C**) defibrillated celluloses obtained from citric acid pretreatment (CA-DFCs; C1–C4 corresponded to citric acid concentrations of 0.2–0.8M, respectively); (**D**) defibrillated celluloses obtained from MW pre-treatment (MW-DFCs; M1–M4 corresponded to MW pre-treatment temperature of 100–160 °C, respectively).

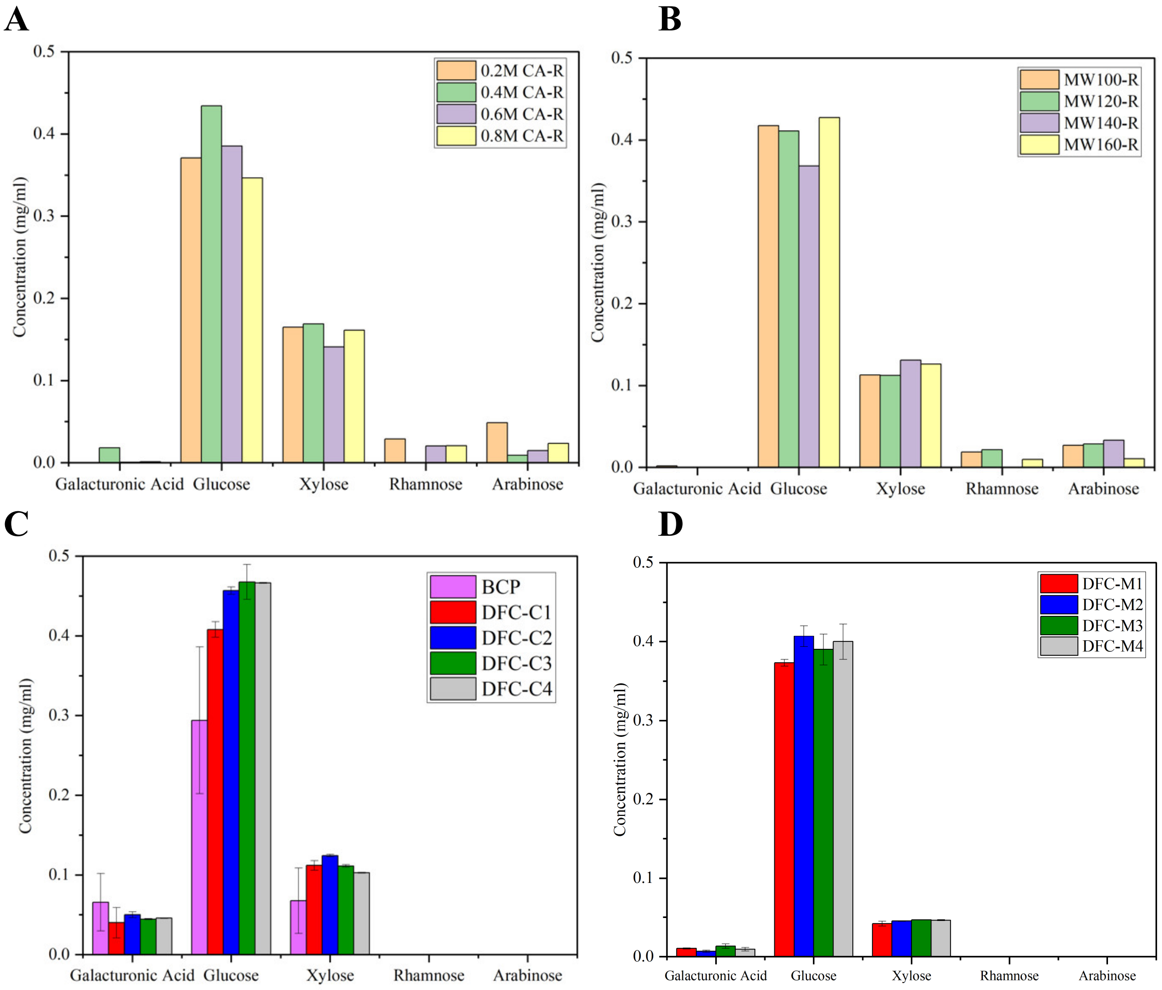

Fig. S2. Carbohydrate and sugar analysis of (**A**) BCP residues after citric acid pretreatment with concentrations of 0.2-0.8M, respectively; (**B**) BCP residues after MW pretreatment with MW temperature of 100-160 °C, respectively; (**C**) BCP and defibrillated celluloses obtained from citric acid pretreatment (CA-DFCs; C1-C4 corresponded to citric acid concentrations of 0.2-0.8M, respectively); (**D**) defibrillated celluloses obtained from MW pre-treatment (MW-DFCs; M1-M4 corresponded to MW pre-treatment temperature of 100-160 °C, respectively).

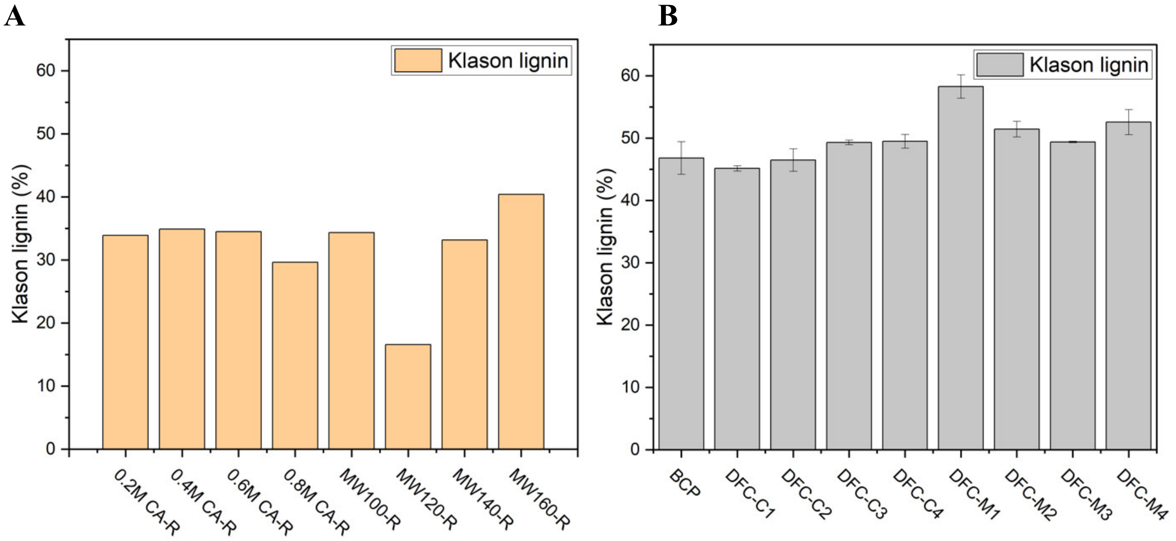

Fig. S3. Klason lignin analysis of (**A**) BCP residues after citric acid and MW pretreatment with citric acid concentrations of 0.2-0.8M and MW temperature of 100-160 °C, respectively; (**B**) BCP, defibrillated celluloses obtained from citric acid pretreatment (CA-DFCs; C1-C4 corresponded to citric acid concentrations of 0.2-0.8M, respectively), and defibrillated celluloses obtained from MW pre-treatment (MW-DFCs; M1-M4 corresponded to MW pretreatment temperature of 100-160 °C, respectively).

A

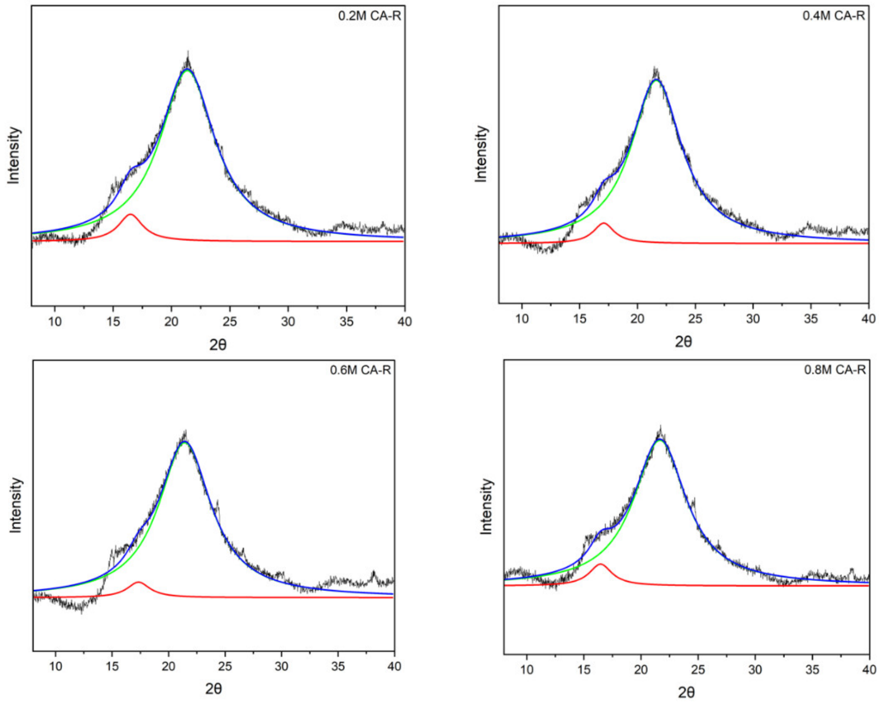

B

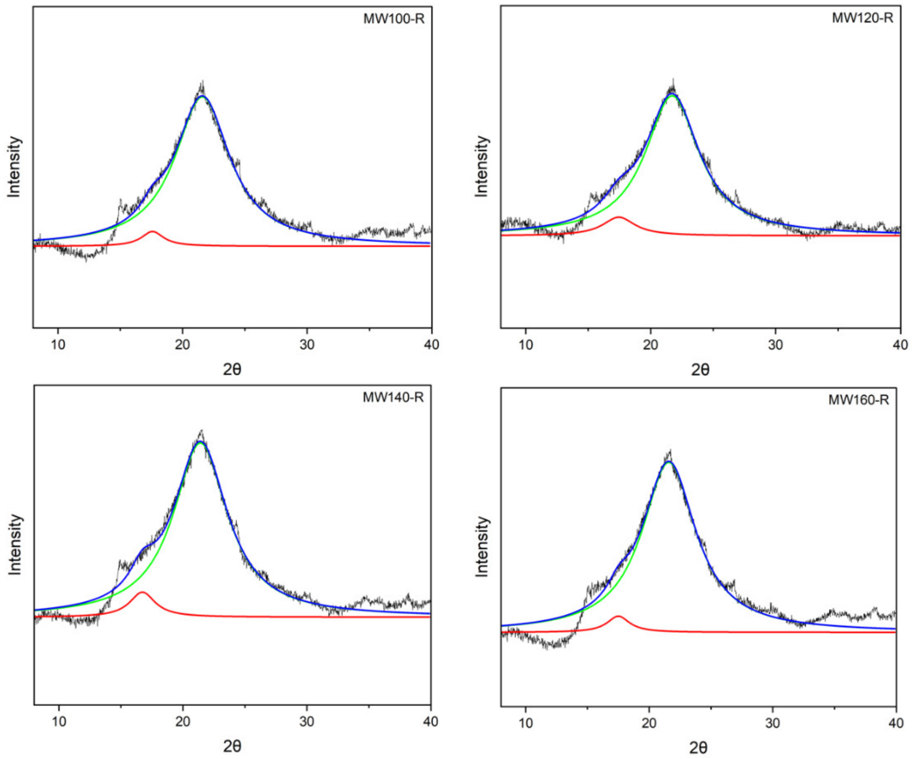

Fig. S4. X-ray diffractograms of (**A**) BCP residues after citric acid pretreatment with concentrations of 0.2-0.8M, respectively; (**B**) BCP residues after MW pretreatment with MW temperature of 100-160 °C, respectively.

A

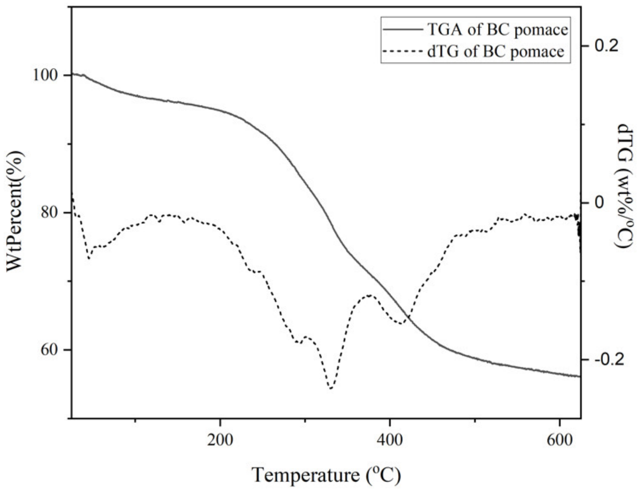

B

C

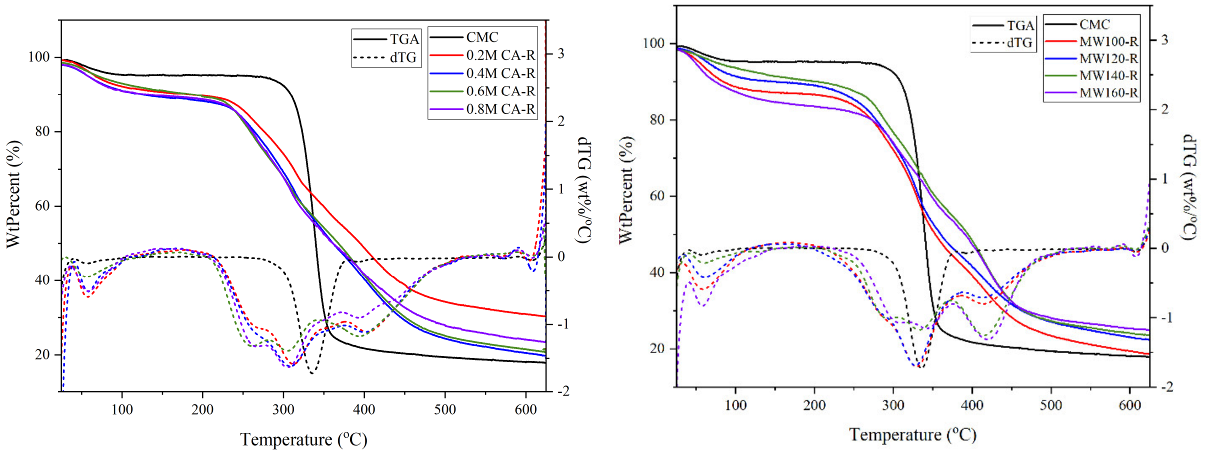

Fig. S5. TGA analysis of (**A**) BCP; (**B**) BCP residues after citric acid pretreatment with concentrations of 0.2-0.8M, respectively; (**C**) BCP residues after MW pretreatment with MW temperature of 100-160 °C, respectively.

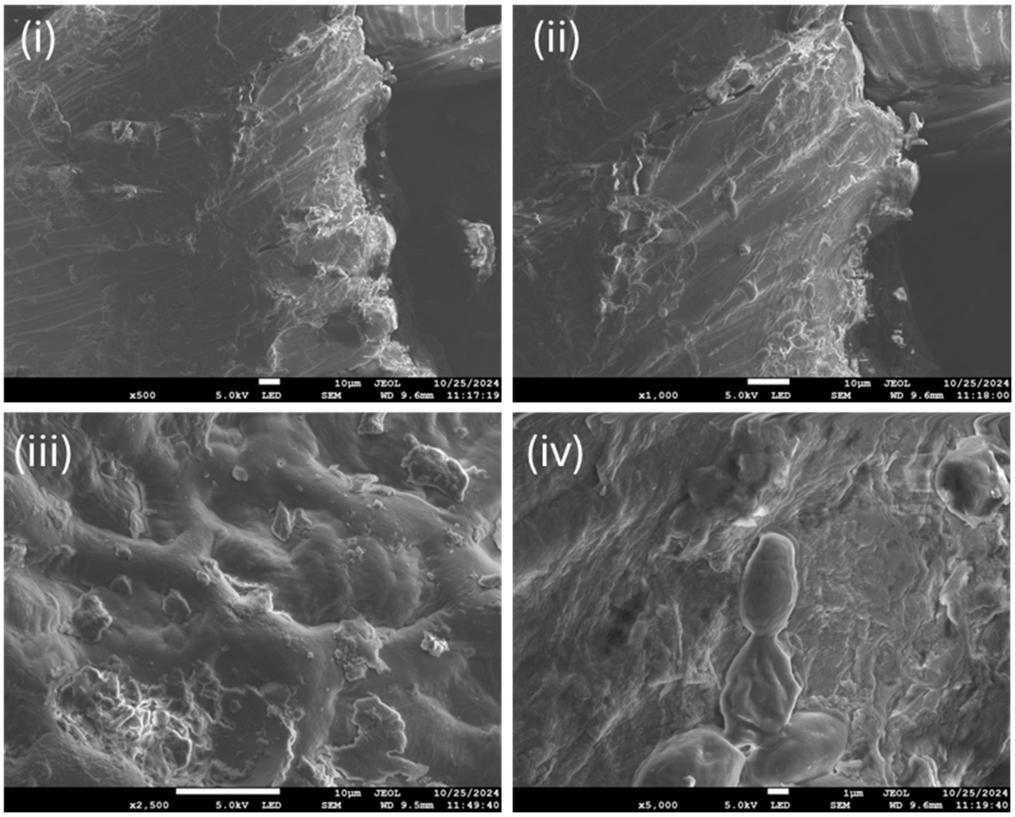

Fig. S6. SEM images of BCP at magnification of (i) 500×; (ii) 1,000×; (iii) 2,500×; (iv) 5,000×.
